# The prognostic significance of PD-L1 and PD-1 expression in patients with nasopharyngeal carcinoma: a systematic review and meta-analysis

**DOI:** 10.1186/s12935-019-0863-5

**Published:** 2019-05-22

**Authors:** Zi-Lu Huang, Shan Liu, Guan-Nan Wang, Shuo-Han Zheng, Shi-Rong Ding, Ya-lan Tao, Chen Chen, Song-Ran Liu, Xin Yang, Hui Chang, Xiao-Hui Wang, Yun-Fei Xia

**Affiliations:** 1 State Key Laboratory of Oncology in South China, Collaborative Innovation Center for Cancer Medicine, Guangdong Key Laboratory of Nasopharyngeal Carcinoma Diagnosis and Therapy, Sun Yat-sen University Cancer Center, 651 Dongfeng Road East, Guangzhou, 510060 People’s Republic of China; 2 Department of Radiation Oncology, Sun Yat-sen University Cancer Center, Guangzhou, People’s Republic of China; 3 Department of Head and Neck Surgery, Sun Yat-sen University Cancer Center, Guangzhou, People’s Republic of China; 4 Department of Pathology, Sun Yat-sen University Cancer Center, Guangzhou, People’s Republic of China

**Keywords:** Nasopharyngeal carcinoma, PD-L1, PD-1, Prognostic, Meta-analysis

## Abstract

**Background:**

Whether PD-L1/PD-1 expression plays a significant role in the prognosis of NPC is still controversial. The present study mainly aimed to investigate the prognostic significance of PD-L1/PD-1 expression in patients with NPC.

**Methods:**

A systematical research was performed in the PubMed, Web of Science, EMBASE, and the Cochrane Library databases up to January 06, 2019. Eighteen studies met eligible criteria were included in the meta-analysis. Quality assessment of included articles was evaluated by Newcastle–Ottawa quality assessment scale (NOS). Pooled hazard ratios (HRs) and their corresponding 95% confidence intervals (95% CIs) were used to elucidated the primary endpoint, overall survival (OS), and the secondary endpoints. Furthermore, the relationship between clinicopathological features of NPC and PD-L1/PD-1 expression was estimated by relative ratios (RRs) and 95% CIs.

**Results:**

A total of 1836 patients from 15 included studies concerning PD-L1 and 678 patients from six studies regarding PD-1 were included in the meta-analysis. Pooled results revealed that PD-L1 expression in NPC did not correlate with OS (HR 1.34 95% CI 0.93–1.93, p = 0.11), DFS (HR 1.82, 95% CI 0.86–3.85, p = 0.12), PFS (HR 1.19, 95% CI 0.46–3.08, p = 0.72), and DMFS (HR 2.26, 95% CI 0.60–8.56, p = 0.23). Meanwhile, no statistically significant differences existed between the expression level of PD-1 in tumor infiltrating lymphocytes (TILs) and the OS in NPC, with the pooled HR 1.29 (95% CI 0.68–2.42, p = 0.44). In subgroup analysis, higher expression of PD-L1 in immune cells correlated with better OS in patients with NPC, with a pooled HR 0.68 (95% CI 0.47–0.99, p = 0.04). Among the clinicopathological features included in our study, we found that the positive expression of PD-L1 in NPC associated with the higher expression of PD-1 (RR 1.25, 95% CI 1.02–1.52, p = 0.03).

**Conclusions:**

Our meta-analysis indicated that higher/positive expression of PD-L1/PD-1 may not serve as suitable biomarkers for the prognosis of NPC, which was not in consistent with some previous studies about the prognostic value of PD-L1/PD-1 in other types of tumors. Despite the positive results in subgroup analysis and study about clinicopathological features, it may still need corroboration of prospective and large-scale studies.

**Electronic supplementary material:**

The online version of this article (10.1186/s12935-019-0863-5) contains supplementary material, which is available to authorized users.

## Background

Nasopharyngeal carcinoma (NPC) is a malignancy with remarkable difference in region distributions, which is of high incidence in the Southeast Asia, the Arctic region, and the North Africa, especially in the Guangdong Province in Southern China [1]. In accordance with the coming era of intensity-modulated radiation therapy (IMRT) and increasing applications of potent chemotherapy, the overall survival and tumor local control rate of patients with nasopharyngeal carcinoma have been considerably improved [2, 3]. Despite the best available treatment, local recurrence and distant metastasis remain to be the main reason for failure after NPC treatment, approximately 5–15% and 15–30%, respectively [4]. Recently, increasing numbers of clinical trials concerning immunotherapy have shown promising effects on patients with nasopharyngeal carcinoma [5, 6]. These studies indicate that the mechanisms of immune evasion exert an enormous function on the pathogenesis of NPC. For these reasons, it would be invaluable for optimizing the treatment in NPC patients that if we could elucidate the relationship between the molecules in immune system and NPC.

As is well known, tumor cells can display immune evasion by activating immune checkpoint molecules. Programmed cell death ligand-1 (PD-L1), an immunoinhibitory molecule, has the function of inducing T-cell-mediated immune tolerance, including anergy and apoptosis, by activating programmed cell death-1 (PD-1) located on the surface of T cells [7]. Therefore, immune therapies targeting PD-1/PD-L1 axis have shown significant anti-tumor effect in some types of solid tumors, including melanoma, non-small-cell lung cancer, and head and neck carcinomas [8].

PD-L1 has been proven to be overexpressed in many types of cancer cells and associated with different clinical outcomes, either better or worse, depending on the categories of tumor [9–12]. Also, there are some controversial about the prognostic value of PD-L1 expression on particular types of cancer, such as breast cancer [13, 14], and nasopharyngeal carcinoma [15–17]. However, there is relatively fewer studies towards the prognostic value of PD-1 expression in patients with cancer. A meta-analysis study demonstrated that the positive expression of PD-1 in TILs correlated with poorer overall survival in patients with epithelial-originated cancer, while the study did not include any data about nasopharyngeal carcinoma [18].

Since the prognostic value of PD-L1 or PD-1 expression in nasopharyngeal carcinoma remains unclear, the aim of the present study was to incorporate all available data using the method of meta-analysis to explore whether different expression status of PD-L1 or PD-1 in patients suffering for nasopharyngeal carcinoma have an effect on their survivals. Also, the correlation between clinicopathological features in patients with NPC and PD-1/PD-L1 expression has been evaluated.

## Methods

### Protocol and registration

This systematic review has been conducted following the “Preferred Reporting Items for Systematic Reviews and Meta-Analyses” (PRISMA) guidelines [19] and the Cochrane Handbook [20]. Also, this review was registered ahead on the online database of International prospective register of systematic reviews (PROSPERO) with the Registration Number of CRD42018109532.

### Search strategy

We performed the literature research in the PubMed, Web of Science, EMBASE, and the Cochrane Library databases for all relevant original articles up to January 06, 2019. The comprehensive search strategies were based on the combinations of the following key words: “programmed cell death-ligand 1, PD-L1, CD274, B7-H1, programmed cell death 1, PD-1, CD279” AND “nasopharyngeal, nasopharynx” AND “carcinoma, tumor, cancer”, with language restricted in English. In addition to the search online, manual search was performed on the reference lists of retrieved articles as well to broaden the search. When it occurs that multiple articles include the same cohort of NPC patients, multiple aspect of evaluation would be carried out in order to decide the final included report.

### Inclusion and exclusion criteria

The eligible criteria were the following: cohort studies were conducted in human with NPC and the diagnosis of NPC was confirmed by pathology; the expression status of PD-1 or PD-L1 was detected: the correlation between PD-1/PD-L1 and overall survival (OS), disease-free survival (DFS), progression-free survival (PFS), or distant-metastasis free survival (DMFS) were elucidated by hazard ratio (HR) and its 95% confidence intervals (95% CI) or Kaplan–Meier curve [21]. Studies were excluded if they met either of the following exclusion criteria: review or basic research; conference abstract or letters; as well as case report or clinical trials.

### Data extraction and quality assessment

Two investigators (Zi-Lu Huang and Shan Liu) independently extracted relevant data from the included studies and summarized it. Any disagreements appeared would be resolved by consulting an adjudicating senior author (YF Xia). The following data were extracted from the eligible studies: name of the first author, year of publication, country where the study was carried out, number of patients, clinicopathological features in different expression of PD-1/PD-L1, cut off value for PD-1/PD-L1 overexpression, detection area, detection methods, prognostic endpoints of interest, statistical analysis approach, and HR and its 95% CI for the endpoints of interest.

The methodological quality of the retrieved articles was evaluated according to the Newcastle–Ottawa quality assessment scale (NOS) for cohort study. The scale is composed of eight items of assessment that can be divided into the following three subgroups: selection, comparability, and outcome, with the subtotal score of 4, 2, and 3, respectively. Finally, a total score of 0–9 was distributed to each eligible study.

### Endpoints of interest and statistical analysis

The primary endpoint of our study was overall survival (OS). If adequate data is available, the primary outcomes would be subdivided in order to make further analysis of subgroups. The secondary endpoints were disease-free survival (DFS), progression-free survival (PFS), distant-metastasis free survival (DMFS), and the clinicopathological factors.

The statistical analysis was performed using Review Manager 5.3 (Cochrane Collaboration, Oxford, UK) and STATA 14. We estimated the prognostic significance of PD-1/PD-L1 expression in NPC by directly using HR and its 95% CI when the original articles reported, otherwise, the Kaplan–Meier curve would be used to obtain HR and its 95% CI using the method provided by Tierney et al. [21]. Statistical heterogeneity between studies was quantified by using the Q test and the I^2^ statistic. The p-value of Q test lower than 0.1 or the statistic of I^2^ higher than 50% was considered that it existed heterogeneity between studies. When it appeared heterogeneity, a random effects model would be chosen to pool the data, otherwise, a fixed effects model would be used. Subgroup analysis and sensitivity analysis were performed to track the origin of the heterogeneity. In this article, we adopted HR > 1 as the benchmark of PD-1/PD-L1 overexpression indicating a poorer outcome. We evaluated the publication bias through the Begg’s and Egger’s test in quantitatively. A two-tailed p value < 0.05 was considered to be statistically significant.

## Results

### Study selection

**Fig. 1:**
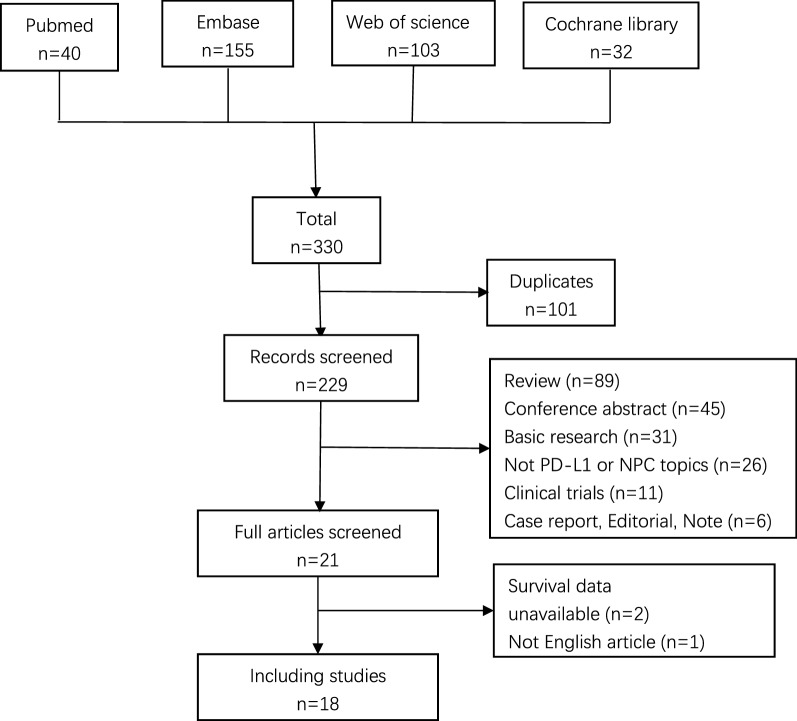
Selection flowchart of the included studies

A total of 330 articles were obtained through the search strategy mentioned above. After removing duplicates, a number of 229 studies left. We performed a screening of title and abstract on the 229 records and 208 records were excluded according to the inclusion and exclusion criterion. The remaining 21 studies were included in the full-text evaluation. Among these, 2 articles didn’t provide available survival data and 1 article was found not written in English. Finally, 18 articles were included in our meta-analysis, including 15 for PD-L1 and 6 for PD-1, severally. There were three studies that not only supplied available data for PD-L1 but also for PD-1. The selection flowchart was displayed in Fig. 1.

### Study characteristics

A total of 1836 patients from 15 included studies concerning PD-L1 [15–17, 22–33] and 678 patients from six studies regarding PD-1 [22, 30, 31, 34–36] were included in the meta-analysis. The publication year of PD-L1 and PD-1 ranged from 2014 to 2018 and 2010 to 2018, respectively. All the included studies were performed in Asia and the majority of these studies were carried out in China. The primary endpoint, OS, was discussed among 11 studies in PD-L1 and six studies in PD-1. The included studies vary in their detection regions of PD-L1/PD-1 expression and the cut-off value for PD-L1/PD-1 positive, which may consequently have an effect on the positive rate of PD-L1/PD-1 overexpression. The quality score differs from 6 to 9 on the basis of the Newcastle–Ottawa quality assessment scale (NOS). The baseline characteristics of included studies are listed in Tables 1 and 2, separately for PD-L1 and PD-1. Among those studies none was found utilized the same cohort of patients.

**Table 1:** Main characteristics of included studies about PD-L1 in this meta-analysis

Author	Year	Origin	Range of year	Categories	No. of patients	Cut off value for PD-L1 positive	No. of positive	Detected area	Endpoints	Detection method	Hazard ratio	Calculation of HRs	Quality score
Cao et al.	2018	China	NA	Primary	108	H-score > 140	18	TC	OS, PFS	FFPE, IHC	K–M curve	Univariate	7
Chan et al.	2017	Hong Kong	2005–2009	primary	161	≥ 1%, ≥ 5%	122, 38	TC or IC	OS, PFS	FFPE, IHC	Report	Univariate and multivariate	7
Chang et al.	2017	Philippines	2008–2011	NA	56	≥ 1%, ≥ 5%	36, 30	TC	OS	FFPE, IHC	Report	Univariate	7
Fang et al.	2014	China	2004–2008	Primary	139	H-score > 35	62	TC and mesenchymal cells	DFS	FFPE, IHC	Report	Univariate	8
Larbcharoensub et al.	2018	Thailand	2007–2012	Primary	114	≥ 5%	81	TC or IC	OS	FFPE, IHC	K–M curve	Univariate	8
Lee et al.	2016	Hong Kong	2005–2009	Primary	104	> 25%	22	TC	OS, PFS, DMFS	FFPE, IHC	Report	Univariate and multivariate	9
Li et al.	2017	China	2009–2015	Primary	120	H-score ≥ 5	54	TC	OS, DFS	FFPE, IHC	K–M curve	Univariate	6
Liu et al.	2018	China	NA	NA	208	≥ 4% (TC), ≥ 2% (IC)	107 (TC), 131 (IC)	TC, IC, TC and IC	OS, DFS	FFPE, IHC	Report	Univariate and multivariate	7
Ono et al.	2018	Japan	2000–2015	Primary	66	≥ 5%	53 (TC), 50 (IC)	TC, IC	OS PFS	FFPE, IHC	Report	Univariate and multivariate	8
Qu et al.	2018	China	NA	NA	96	> 10%	28	TC	DMFS	FFPE, IHC	K–M curve	Univariate	7
Zhang et al.	2015	China	NA	NA	139	H-score > 35	58	Tumor tissue	DFS	FFPE, IHC	K–M curve	Univariate	7
Zheng et al.	2017	China	2010–2012	Primary	85	Score ≥ 3	29	TC	OS, DMFS	FFPE, IHC	Report	Multivariate	7
Zhou et al.	2017	China	2001–2013	Recurrence	132	H-score > 190	88	TC	OS	FFPE, IHC	Report	Multivariate	9
Zhou et al. [2]	2017	China	2010–2012	Primary	99	H-score ≥ 155	61	TC	OS	FFPE, IHC	Report	Multivariate	9
Zhu et al.	2017	China	1991–2000	Primary	209	≥ 5%	68 (TC), 98 (IC)	TC, IC	OS, DFS	FFPE, IHC	Report	Univariate and multivariate	8

DFS: disease-free survival; DMFS: distant metastasis-free survival; FFPE: Formalin-Fixed and Paraffin-Embedded; HRs: hazard ratios; IC: immune cells; IHC: immunohistochemistry; K–M curve: Kaplan–Meier curve; NA: not available; No: number; OS: overall survival; PFS: progression-free survival; TC: tumor cells

**Table 2:** Main characteristics of included studies about PD-1 in this meta-analysis

Author	Year	Origin	Range of year	Categories	No. of patients	Cut off value for PD-1 positive	No. of positive	Detected area	Endpoints	Detection method	Hazard ratio	Calculation of HRs	Quality score
Cao et al.	2018	China	NA	Primary	108	H-score > 0	41	TIL	OS	FFPE, IHC	Report	Multivariate	7
Hsu et al.	2010	China	2003–2004	Primary	46	> Median expression rate: 27.8% (intratumoral CD8), > median expression rate: 14.5% (intratumoral CD4)	23, 23	Intratumoral CD8, intratumoral CD4	OS	FFPE, IHC	Report	Multivariate	7
Lu et al.	2018	China	2007–2012	Primary	197	PD-1 staining intensity 2 in > 5% of TILs	96	TIL	OS	FFPE, IHC	K–M curve	Univariate	7
Tang et al.	2017	China	NA	NA	96	NA	36	TIL	OS	FFPE, IHC	K–M curve	Univariate	6
Zhou et al.	2017	China	2001–2013	Recurrence	132	NA	50	TIL	OS	FFPE, IHC	Report	Univariate	9
Zhou et al. [2]	2017	China	2010–2012	Primary	99	PD-1 staining intensity 2 in > 5% of TILs	44	TIL	OS	FFPE, IHC	K–M curve	Univariate	9

FFPE: Formalin-Fixed and Paraffin-Embedded; HRs: hazard ratios; IHC: immunohistochemistry; K–M curve: Kaplan–Meier curve; NA: not available; No: number; OS: overall survival; TIL: tumor infiltrating lymphocytes

### Synthesis of results

**Fig. 2:**
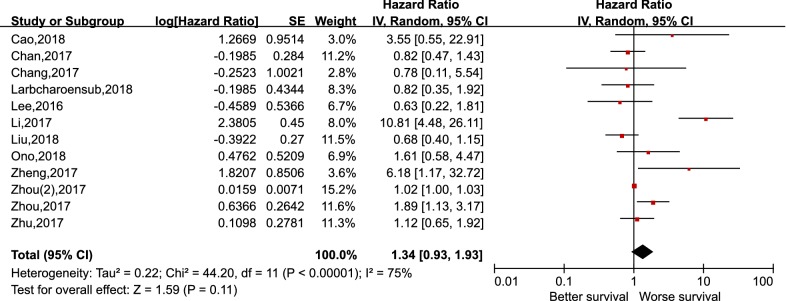
Forest plot for overall survival (OS) of PD-L1 high expression in nasopharyngeal carcinoma

**Fig. 3:**
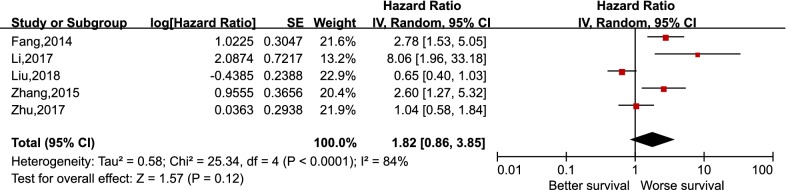
Forest plot for disease-free survival (DFS) of PD-L1 high expression in nasopharyngeal carcinoma

**Fig. 4:**
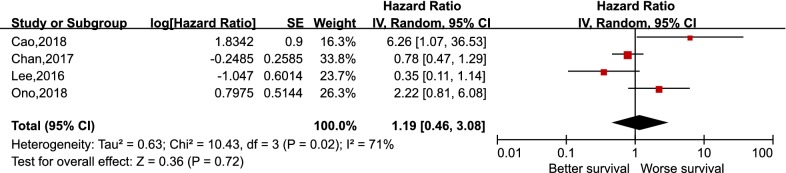
Forest plot for progression-free survival (PFS) of PD-L1 high expression in nasopharyngeal carcinoma

**Fig. 5:**
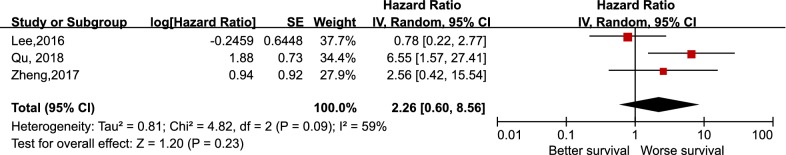
Forest plot for distant-metastasis free survival (DMFS) of PD-L1 high expression in nasopharyngeal carcinoma

**Fig. 6:**
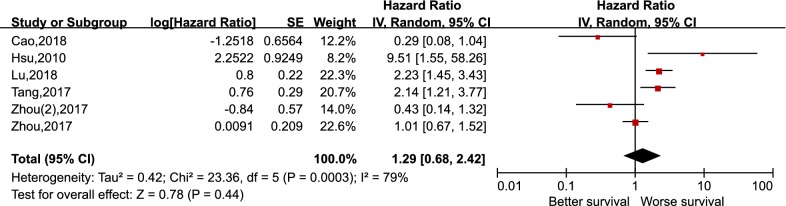
Forest plot for overall survival (OS) of PD-1 high expression in nasopharyngeal carcinoma

As depicted in Fig. 2, pooling the data from 12 studies that assessed the prognostic value of PD-L1 expression in NPC showed no significant association between PD-L1 expression and OS, with the pooled HR 1.34 (95% CI 0.93–1.93, p = 0.11). Furthermore, a random-effects model was adopted for the reason that a significant heterogeneity was calculated among the studies (I^2^ = 75%, p < 0.000). For the secondary endpoints, no significant correlation was observed between the expression of PD-L1 and DFS (HR 1.82, 95% CI 0.86–3.85, p = 0.12), PFS (HR 1.19, 95% CI 0.46–3.08, p = 0.72), and DMFS (HR 2.26, 95% CI 0.60–8.56, p = 0.23) likewise (Figs. 3, 4 and 5). It was demonstrated in Fig. 6 that no statistically significant differences existed between the expression level of PD-1 in tumor infiltrating lymphocytes and the OS in NPC, with the pooled HR 1.29 (95% CI 0.68–2.42, p = 0.44). Meta-analysis concerning the prognostic value of PD-1 in NPC was not performed in the secondary endpoints, DFS and PFS, due to the lack of adequate studies.

### Sensitivity analysis and subgroup analysis

The results of sensitivity analysis and subgroup analysis on the correlation of PD-L1 expression and OS in NPC were displayed on Table 3. Sensitivity analysis was performed and we found the most significant heterogeneity originated from the study of Li et al. [26]. After remove it, I^2^ decreased to 40% and p value of heterogeneity increased to 0.08, with the pooled HR 1.07 and its 95% CI 0.84–1.36. Subgroup analysis was carried out on the cut-off value of PD-L1 positive, and the results indicated that no significant correlation existed between the cut-off value for PD-L1 positive (both ≥ 5% and ≥ 1%) and OS in NPC patients. It was noteworthy that heterogeneity was not as remarkable as the pooled data before, when we conducted the subgroup analysis based on the cut-off value for PD-L1 positive. (I^2^ = 0% and p = 0.77, I^2^ = 0% and p = 0.59, particularly.) Subgroup analysis was also performed on the basis of detection area. Compared with higher expression of PD-L1 in tumor cells, an advantage of overall survival was shown in the set of PD-L1 lower expression. However, the difference was not statistically significant (HR 1.55, 95% CI 0.99–2.42, p = 0.06). A statistical difference was observed between the PD-L1 expression in immune cells and OS in patients of NPC, with a pooled HR 0.68 (95% CI 0.47–0.99, p = 0.04) and a fixed-effects model was used. We found no significant difference in subgroup analysis of the estimation and calculation methods of HRs.

**Table 3:** Subgroup analysis of included studies about PD-L1 and OS in this meta-analysis

Subgroup	No. of studies	No. of patients	HR (95% CI)	p value	Heterogeneity		Statistical model used
					I^2^ (%)	p value	
Cut-off value for PD-L1 positive							
≥ 5%	5	606	0.98 (0.70, 1.35)	0.89	0	0.77	Fixed
≥ 1%	2	217	0.83 (0.50, 1.37)	0.47	0	0.59	Fixed
Detection area							
Tumor cells	10	1561	1.55 (0.99, 2.42)	0.06	79	0.000	Random
Immune cells	3	483	0.68 (0.47, 0.99)	0.04	0	0.57	Fixed
TC or IC	2	275	0.82 (0.51, 1.31)	0.40	0	1.00	Fixed
Calculation of HRs							
Multivariate	9	1558	1.40 (0.93, 2.10)	0.11	81	0.000	Random
Univariate	9	1146	1.06 (0.58, 1.91)	0.86	79	0.000	Random
Estimation method of HRs							
Reported	10	1604	1.37 (0.92, 2.03)	0.12	79	0.000	Random
K–M curve	3	342	3.12 (0.49, 19.83)	0.23	88	0.000	Random

HRs: hazard ratios; IC: immune cells; K–M curve: Kaplan–Meier curve; No: number; OS: overall survival; TC: tumor cells; 0.000, P < 0.001

### Clinicopathological features

The relationship between clinicopathological factors and the expression level of PD-L1/PD-1 in NPCs was summarized in Table 4a and b. The features included in this article were mainly as follows: gender, TNM stage, tobacco or alcohol use, Epstein–Barr virus (EBV) status, pathologic types. Among these characteristics, no statistical difference was discovered. However, we found that the positive expression of PD-L1 in NPCs correlated with the higher expression of PD-1 (RR 1.25, 95% CI 1.02–1.52, p = 0.03).

**Table 4:** The relationship between clinicopathological factors and the expression level of (a) PD-L1 in NPCs, (b) PD-1 in NPCs

Clinicopathological factors	RR	95% CI	p value	Heterogeneity		No. of studies	Statistical model used
				I^2^ (%)	p value		
(a)							
Gender: male	1.02	(0.96, 1.09)	0.45	0	0.70	13	Fixed
T stage ≥ 3	1.00	(0.89, 1.13)	0.88	42	0.07	11	Random
N stage ≥ 2	0.98	(0.87, 1.10)	0.77	34	0.12	11	Fixed
Metastasis	0.83	(0.52, 1.33)	0.45	0	0.45	4	Fixed
Stage III or IV	1.07	(0.99, 1.15)	0.08	38	0.12	9	Fixed
Undifferentiated	0.99	(0.84, 1.18)	0.94	54	0.07	5	Random
Tobacco use: smoker	0.91	(0.78, 1.07)	0.24	0	0.43	8	Fixed
Alcohol use: drinker	0.73	(0.49, 1.09)	0.13	0	0.63	2	Fixed
EBV positive	1.03	(0.93, 1.15)	0.54	29	0.24	2	Fixed
(b)							
Gender: male	1.04	(0.96, 1.13)	0.35	0	0.99	6	Fixed
T stage ≥ 3	0.93	(0.81, 1.07)	0.29	0	0.66	4	Fixed
N stage ≥ 2	1.11	(0.95, 1.29)	0.20	0	0.97	5	Fixed
Stage III or IV	1.02	(0.94, 1.10)	0.65	29	0.23	5	Fixed
Tobacco use: smoker	1.10	(0.85, 1.43)	0.46	0	0.85	3	Fixed
PD-L1 positive	1.25	(1.02, 1.52)	0.03	0	0.80	4	Fixed

RR: relative risk; CI: confidence interval; No: number; NPC: nasopharyngeal carcinoma; EBV: Epstein–Barr virus

### Publication bias

**Fig. 7:**
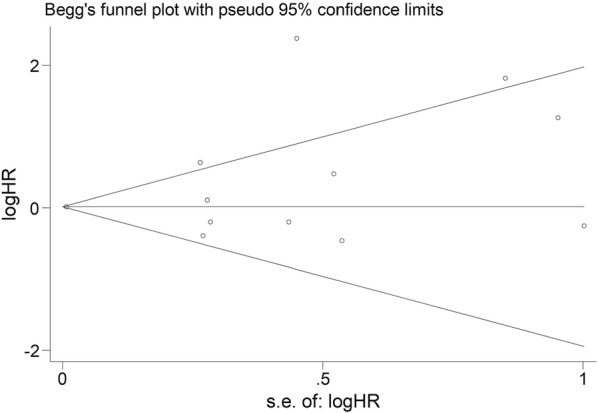
Begg’s funnel plot for assessing publication bias for the impact of PD-L1 expression on OS in patients of nasopharyngeal carcinoma

**Fig. 8:**
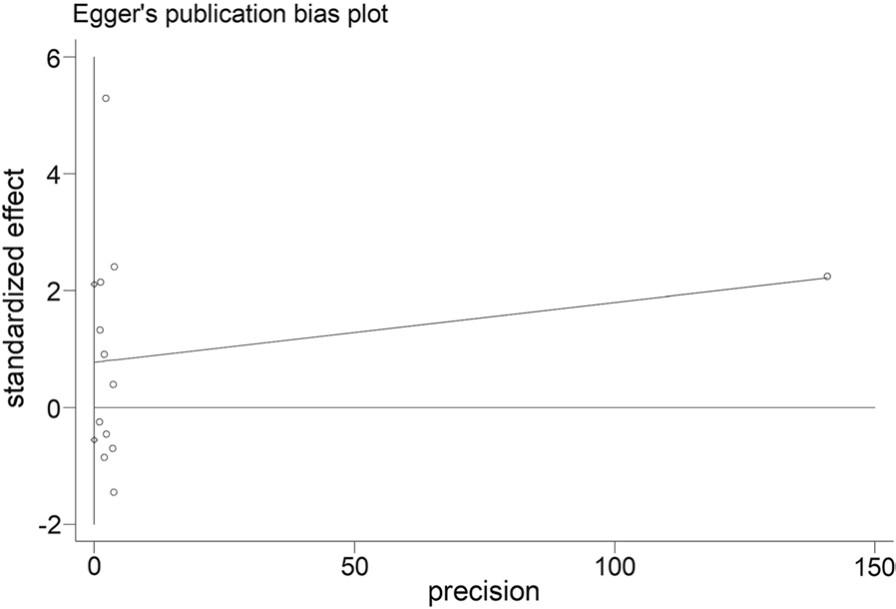
Egger’s plot for assessing publication bias for the impact of PD-L1 expression on OS in patients of nasopharyngeal carcinoma

In our meta-analysis, the publication bias was assessed by Begg’s funnel plot and Egger’s test. 12 articles concerning the impact of PD-L1 expression on OS in patients of NPC was included in these two tests. No evidence observed in Begg’s funnel plot (p = 0.273) (Fig. 7, Additional file 1) and Egger’s test (p = 0.224) (Fig. 8, Additional file 1) showed publication bias among these studies.

## Discussion

This meta-analysis of 18 retrospective cohort studies including 15 articles covering PD-L1 and six studies concerning PD-1, a total of 1836 patients, comparing different expression level of PD-L1/PD-1 and the prognosis of NPC showed that no statistical significance was found between higher/positive expression of PD-L1/PD-1 and the prognosis of NPC. Among the subgroup analysis, when the detection area of PD-L1 was the immune cells, a higher expression of PD-L1 displayed better overall survival in NPC. Observing all the clinicopathological characteristics, only the expression of PD-L1 was found correlate with PD-1, and no other clinicopathological features was related with the expression of PD-L1/PD-1.

PD-1, known as an inhibitory receptor, which is mainly expressed on activated T lymphocytes, but also can be observed on B cells and NK cells [37]. PD-L1 as well as PD-L2, one of the ligands for PD-1 receptor, belonging to B7 family is found expressed on PD-L1 contributes to the tolerance and impairment of the immune system [38]. The tumor microenvironment makes contribution to the upregulation of PD-1 expression in TILs, which leads to the impairment of antitumor immune. Since the upregulated PD-1 in TILs will result in an exhausted phenotype and impaired function of T cells in TILs [39]. At the same time, it is known that PD-L1 has been discovered expressing on many types of tumor lines, and its combination with PD-1 in TILs serves as one of the pivotal mechanism of modulating tumor cells with immunogenicity to escape from the surveillance of the host immune system [40].

On account of various of tumor types and therapeutics, the prognostic value of PD-L1/PD-1 in tumor has not reach a consensus. In a meta-analysis about solid tumors included 61 articles showed that the overexpression of PD-L1 suggested a worse prognosis [41]. However, when it comes to some specific types of tumors, the results were not the same. For instance, a study pooled 11 articles revealed that the expression of PD-L1 had no statistical correlation with the prognosis of patients with oral squamous cell carcinoma, whatever in OS, DFS or disease-specific survival (DSS) [42]. In addition, another meta-analysis concerning osteosarcoma involving eight original studies indicated that the overexpression of PD-L1/PD-1 significantly related with a higher incidence of metastasis and total mortality risk [43].

In our study, the prognostic value of PD-L1 expression in NPC was reported in 15 eligible studies. A previous study regarding the correlation of PD-L1 expression and head and neck carcinoma (HNC) included 17 original articles totally, while only 2 of them are about NPC [44]. The conclusions drawn from the present study are something different from the previous one. The meta-analysis study about HNC demonstrated that no association was found between PD-L1 expression and the prognosis of HNC, however, the subgroup analysis indicated that in the group of Asia regions/countries a poorer OS in HNC may correlated with positive expression of PD-L1. Compared with the previous study, original studies included in our meta-analysis were all done in Asia regions/countries and the pooled data revealed no statistical difference between the expression level of PD-L1 and prognosis of NPC. It may be because nasopharyngeal carcinoma and non-nasopharyngeal head and neck cancer vary in their clinicopathological features and therapeutics.

The results of another recently published meta-analysis elucidating the prognostic value of some immune checkpoints in head and neck cancer were completely different from our meta-analysis and the previous study of head and neck that we just mentioned above [45]. The analysis demonstrated that PD-L1 expression higher indicated better OS in HNC, especially in NPC (p = 0.01). While, by comparison with our study, several obvious distinctions deserve to be concerned. First, we found that it only included seven studies in the meta-analysis of PD-L1 expression in NPC about OS, by contrast, the present study included 12 eligible studies to pool the data regarding the impact of PD-L1 expression in OS, containing the previous seven studies. Second, some details of the data (HR and 95% CI) adopted were nuance. Since a few of survival data were derived from Kaplan–Meier curve, data deviation was inevitable. Also, when the eligible original articles provided two or more alternative survival data, choice among researchers could not reach unanimous unless prior discussion. For example, the study of Ono et al. [27] offered the results of both univariate and multivariate, however, Jia et al. [45] utilized the univariate one and our meta-analysis chose multivariate one. Similarly, Chang et al. [23] supplied survival data in accordance with two PD-L1 cut-off value, while Jia et al. [45] used the 1% cut-off value and we adopted the most common one, 5% cut-off value, to pool data.

Among all the original reports eligible in our study, some of the studies demonstrated that relatively higher expression of PD-L1 or PD-1 in NPC predicts worse prognosis [22, 24, 26, 28, 29, 31, 34–36]. The most common and convincing explanation for the result was the interactions of PD-1 and PD-L1 would lead to immune suppression and promote tumor progression [38, 40]. None of the multivariate analysis results in eligible studies approved for the main conclusion drew in Jia et al. [45]. Remaining original studies declared no statistically significant difference in the expression of PD-1 or PD-L1 and the prognosis of NPC, which were consistent with our conclusion.

Interferon-gamma (IFN-γ) has been shown to be detected in tumor cells with PD-L1 positive and TILs, and act as a primary inducer for PD-L1 expression, while it could not be detected in PD-L1 negative tumor cells [46]. Therefore, PD- L1 expression may not directly result in immune evasion and it may represent an outcome of on-going immune system triggers immune inhibition by producing IFN-γ or other cytokines [25, 46]. In the tumor microenvironment of NPC, the condition of infiltrating lymphocytes is more complexity, on account of the high infectious rate of Epstein–Barr virus (EBV) in NPC [47]. Sustainably exposure in viral contributes to more active inflammatory reactions resulting in the expression of PD-L1 [48, 49]. It can be inferred that the expression of PD-L1 in NPC may just reflect the inflammatory response in the tumor microenvironment, which may need further research to identify. The expression of PD-1 in TILs have been shown to be dynamically changed in conformity to the status of immune system and tumor microenvironment, which may illustrate the undefinition of the prognostic value of PD-1 expression in NPC [30, 50]. Recently, more and more clinical trials are focusing on immunotherapy in HNC, especially anti-PD-L1/PD-1 antibody in NPC. Hsu et al. [5] reported a phase Ib clinical trial about anti-PD-1 monoclonal antibody, pembrolizumab, in NPC with PD-L1 positive, and revealed its antitumor activity and manageable adverse events. Another phase I trials concerning anti-PD-1 antibody, camrelizumab, in NPC also derives positive results [51]. Colevas et al. [52] conducted phase Ia trials regarding anti-PD-L1, atezolizumab, in HNC, and get encouraging antitumor activity regardless of PD-L1 expression. Since increasing researchers are concerning about the immunotherapy of anti-PD-L1/PD-1 antibody in NPC, it is worthwhile to figure out the role of PD-L1/PD-1 plays in the tumor microenvironment and prognosis of NPC.

Our meta-analysis performed sensitivity analysis on the outcome of OS regarding PD-L1 by cut-off value, detection area, calculation of HRs, and estimation method of HRs. Though we found that higher expression of PD-L1 in immune cells correlated with better OS, it only included three studies evoking the conclusion may not be robust. When we exclude the study of Li et al. [26], heterogeneity decreased most obviously compared with deleting other single studies. We speculated that it may because of its unique cut-off value for PD-L1 and the estimation of HR was based on Kaplan–Meier curve. Furthermore, we discovered that its quality score was the lowest. However, excluding the study did not influence the main conclusion we drew. We failed to perform subgroup analysis among studies about PD-1, due to the deficiency of related original studies, which is a limitation of our study. Another limitation in our analysis is that the heterogeneity is obvious among studies, and it may originate from varied cut-off value for PD-L1 positive based on the subgroup analysis and summarization of main characteristics of eligible studies. The cut-off value for PD-L1/PD-1 positive in tumor microenvironment may need further discussion to determine suitable world-wide criterions in accordance with different types of tumors.

## Conclusions

Our study revealed that the expression level of PD-L1 or PD-1 may not act as a useful predict biomarkers for the prognosis of NPC. The positive results obtained in subgroup analysis and clinicopathological analysis need further studies to be confirmed.

## Additional file

**Additional file 1.** The data of publication bias test.

## Data Availability

The databases analyzed during the current study are available.

## Abbreviations

CI: confidence interval
DFS: disease-free survival
DMFS: distant metastasis-free survival
EBV: Epstein–Barr virus
FFPE: Formalin-Fixed and Paraffin-Embedded
HRs: hazard ratios
IC: immune cells
IFN-γ: interferon-gamma
IHC: immunohistochemistry
K–M curve: Kaplan–Meier curve
NA: not available
No: number
NOS: Newcastle–Ottawa quality assessment scale
NPC: nasopharyngeal carcinoma
OS: overall survival
PD-1: programmed cell death-1
PD-L1: programmed cell death ligand-1
PFS: progression-free survival
TC: tumor cells
TILs: tumor-infiltrating lymphocytes
