# Supplementary material for: The prognostic significance of PD-L1 and PD-1 expression in patients with nasopharyngeal carcinoma: a systematic review and meta-analysis
Source: Cancer Cell Int. 2019 May 22;19:141. doi: 10.1186/s12935-019-0863-5 (PMC6530183; doi:10.1186/s12935-019-0863-5)
Supplement: Supplementary file 1 — Additional file 1. The data of publication bias test. [file 12935_2019_863_MOESM1_ESM.docx]

Begg's Test

adj. Kendall's Score (P-Q) = 16

Std. Dev. of Score = 14.58

Number of Studies = 12

z = 1.10

Pr > |z| = 0.273

z =1.03 (continuity corrected)

Pr > |z| = 0.304 (continuity corrected)

Egger's test

------------------------------------------------------------------------------

Std_Eff | Coef. Std. Err. t P>|t| [95% Conf. Interval]

-------------+----------------------------------------------------------------

slope | .0102064 .0146659 0.70 0.502 -.0224712 .0428841

bias | .7750279 .5974077 1.30 0.224 -.5560794 2.106135

------------------------------------------------------------------------------
